# An Investigation of the Effects of α‐ and β‐Frequency Neural Entrainment Using tACS on Phase‐Aligned TMS‐Evoked Corticospinal Excitability

**DOI:** 10.1002/brb3.70876

**Published:** 2025-09-30

**Authors:** Aikaterini Gialopsou, Stephen R. Jackson

**Affiliations:** ^1^ School of Psychology University of Nottingham, University Park Nottingham UK; ^2^ Sir Peter Mansfield Imaging Centre, School of Physics and Astronomy University of Nottingham, University Park Nottingham UK; ^3^ Institute of Mental Health, School of Medicine University of Nottingham, University Park Nottingham UK

**Keywords:** α‐tACS, β‐tACS, corticospinal excitability, inter‐trial variability, transcranial alternating current stimulation (tACS) phase‐aligned transcranial magnetic stimulation (TMS)

## Abstract

**Purpose:**

Deep brain stimulation (DBS) is an effective treatment for many brain disorders (e.g., Parkinson's disease), has a favorable adverse effect profile, and can be particularly effective for individuals with treatment‐resistant symptoms. DBS is, however, inaccessible for most individuals, is extremely expensive, and is not considered suitable for children and adolescents. For these reasons, noninvasive alternatives to DBS, such as transcranial magnetic stimulation (TMS), are increasingly being sought to treat brain health conditions. Unfortunately, current TMS approaches exhibit large intra‐ and inter‐subject variability in their efficacy, which limits their use clinically. One likely reason for this is that TMS is invariably delivered without reference to ongoing brain activity (i.e., open loop).

**Methods:**

We propose that the efficacy of stimulation might be improved, and the variability of its effects reduced, if stimulation could be synchronized with ongoing brain activity. To investigate this, we used transcranial alternating current stimulation (tACS) to induce entrainment of brain activity at two frequencies (*α* = 10 Hz and *β* = 20 Hz), and we delivered single‐pulse TMS that was temporally aligned with the phase of each tACS oscillation. To investigate the effects of tACS‐phase‐aligned TMS, we measured motor‐evoked potentials (MEPs).

**Findings:**

Our findings confirm that for α‐ and β‐tACS, both corticospinal excitability and inter‐trial variability varied as a function of tACS phase. Importantly, however, the tACS phase angle that produced maximum TMS‐evoked excitability was different for α‐ and β‐tACS, coinciding with the negative peak (trough) for α‐tACS and the positive peak (peak) for β‐tACS.

**Conclusion:**

These findings confirm that aligning noninvasive brain stimulation to ongoing brain activity may increase the efficacy of TMS and reduce the variability of its effects. However, our results illustrate that the optimal phase of the tACS cycle at which to deliver TMS may vary for different tACS frequencies.

## Graphical abstract

**Effects of alpha (10 Hz) and beta (20 Hz) tACS on corticospinal excitability with phase‐targeted TMS**: Both frequencies modulate excitability and inter‐trial variability in a frequency‐ and phase‐specific manner, suggesting that aligning stimulation with ongoing brain rhythms may enhance TMS efficacy and reliability.

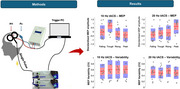

**Abbreviations**
M1: primary motor area
MEP: motor‐evoked potential
Pz: midline parietal
tACS: transcranial alternating current stimulation
TMS: transcranial magnetic stimulation

## 1 Introduction

Deep brain stimulation (DBS) is a surgical procedure in which implanted electrodes deliver high‐frequency electrical stimulation to a targeted brain area to modulate dysfunctional brain network activity. Importantly, the mode‐of‐action of DBS would appear to depend upon neural entrainment, as studies have shown that during DBS neural firing rates become strongly phase‐locked to the DBS pulse trains (McCairn et al. 2013).

DBS is typically used only with treatment‐resistant adults, is very expensive, is not suitable for children or adolescents, and is restricted in its availability. For this reason, noninvasive brain stimulation alternatives to DBS are being sought as these may have the potential to widen access to safe and effective brain stimulation treatments for a broad range of neurological and psychiatric conditions.

Noninvasive brain stimulation approaches, such as transcranial magnetic stimulation (TMS), are increasingly being used to treat brain health conditions such as depression (Perera et al. 2016). However, current TMS approaches have a number of limitations that may limit their use clinically. First, they cannot be used to directly stimulate deep brain areas that may play a role in brain disorders, although they may be effective indirectly. Second, the effects of TMS are often highly variable (Hamada et al. 2013), exhibiting considerable intra‐ and inter‐subject variability, which limits their use clinically.

One likely contributing factor for the variability observed for noninvasive brain stimulation approaches, such as TMS, is that current approaches most often adopt an open‐loop, one‐size‐fits‐all, methodology in which individuals each receive identical stimulation, without reference to ongoing brain activity. However, recent evidence strongly indicates that the efficacy of TMS is increased by adopting a closed‐loop control strategy, in which stimulation is time‐locked to ongoing physiological events within the brain (Zrenner et al. 2018).

Cortical activity is characterized by neural oscillations that are thought to reflect synchronized neural firing. These oscillations can be measured using electroencephalography (EEG) or magnetoencephalography (MEG) and reflect spontaneous fluctuations in brain state that are characterized by differences in cortical activity. Specifically, alterations in cortical excitability are shown to be associated with the instantaneous phase of cortical oscillations (Berger et al. 2014). This may indicate a functional role for brain oscillations in organizing patterns of neural firing in the brain. In the motor cortex, changes in power in α‐band (8–13 Hz) and β‐band (14–30 Hz) oscillations are associated with the preparation and initiation of voluntary movements (Jenkinson and Brown 2011; Combrisson et al. 2017; Miller et al. 2012).

Zrenner et al. (2018) used real‐time, EEG‐triggered, TMS to demonstrate a dependence between TMS‐induced alterations in corticospinal excitability and the phase of the cortical α‐rhythm. They reported that larger motor‐evoked potential (MEP) amplitudes were elicited when TMS was triggered to coincide with the negative peak (trough) of the α‐rhythm, compared to when it was aligned with the positive peak (Zrenner et al. 2018). See also Hussain et al. (2019), Ozdemir et al. (2022). Consistent with this finding, functional magnetic resonance imaging (fMRI) studies have demonstrated that sensorimotor activity (as measured by fMRI BOLD signal) is negatively correlated with the sensorimotor EEG α‐rhythm (Yin et al. 2016).

Wischnewski et al. (2022) also used real‐time, EEG‐triggered TMS to investigate the relationship between TMS‐induced increases in corticospinal excitability and the phase of the cortical α‐ or β‐rhythm. They targeted four phases (0°, 90°, 180°, and 270°) of the α‐ and β‐rhythms with suprathreshold single‐pulse TMS to the primary motor cortex. They reported phase‐dependent modulation of TMS‐induced corticospinal excitability for both the α‐ and β‐rhythm. Importantly, they found that the relationship between EEG phase and TMS‐induced corticospinal output differed for the α‐ or β‐rhythm. MEPs were largest when TMS was aligned with the trough and rising phase of the α‐rhythm compared to when TMS was aligned with either the peak or falling phase. By contrast, for the β–rhythm, MEPs were largest when TMS was aligned with the peak and falling phase of the β‐rhythm.

Studies, such as that reported by Zrenner et al. (2018) and Wischnewski et al. (2022), demonstrate that closed‐loop control of noninvasive brain stimulation can be achieved by temporally aligning stimulation with the phase of brain oscillations measured and analyzed in real‐time using EEG. However, the real‐time analysis of EEG and the accurate temporal prediction of instantaneous oscillatory phase are technically challenging. An alternative, and arguably simpler, method for delivering noninvasive brain stimulation in phase with neural activity may be through neural entrainment.

As noted before, the mode‐of‐action of DBS depends upon neural entrainment as during DBS neural firing becomes strongly phase‐locked to the frequency of DBS pulse trains (McCairn et al. 2013). Importantly, entrainment of neural activity can also be achieved noninvasively using transcranial alternating current stimulation (tACS) (Vogeti et al. 2022). tACS involves applying a weak sinusoidal electrical current between two or more electrodes attached on the scalp and is thought to induce entrainment of neural activity (in phase with the frequency of the tACS stimulation) and spike‐timing‐dependent synaptic plasticity (STDP), leading to stimulation aftereffects (Vogeti et al. 2022). Studies in non‐human primate demonstrate that tACS can induce (a) phase‐entrainment of neural firing (Krause et al. 2019), (b) dose‐dependent increases in neural bursting (Johnson et al. 2020); and (c) cell‐class‐specific entrainment of spiking activity (Lee et al. 2024). Importantly, these studies indicate that although tACS induces phase‐dependent entrainment of neural firing, it does not increase the neural spiking rate (Krause et al. 2019). Studies in humans have demonstrated that β‐frequency tACS can be used successfully to entrain cortical sensorimotor activity and as a result slow the execution of voluntary movement (Pogosyan et al. 2009). Furthermore, recent advances in the methods used to deliver tACS have shown that the modulation of deep brain areas, such as the hippocampus or striatum, is possible using tACS (Krause et al. 2019) or by using a variant of tACS known as temporal interference stimulation (TIS) (Grossman et al. 2017; Violante et al. 2023). It is suggested that tACS might be used to normalize neural “noise” in maladaptive brain networks linked to neurological disease (Krause et al. 2019) and open up the possibility of developing noninvasive alternatives to current surgical DBS procedures.

In the current study, we used α‐ and β‐frequency tACS to entrain cortical motor oscillations and delivered single pulses of above‐threshold TMS to coincide with the peak (0°), falling phase (90°), trough (180°), or rising phase (270°) of the α‐ or β‐phase cycle. TMS‐evoked corticospinal excitability was measured using electromyography (EMG). We hypothesized that (a) there would be phase‐dependent differences in the magnitude of TMS‐evoked MEPs; (b) there would be phase‐dependent differences in the inter‐trial variability of MEP amplitudes; and (c) phase‐dependent effects might be different for tACS entrainment at α and β frequencies.

## 2 Materials and Methods

**FIGURE 1:**
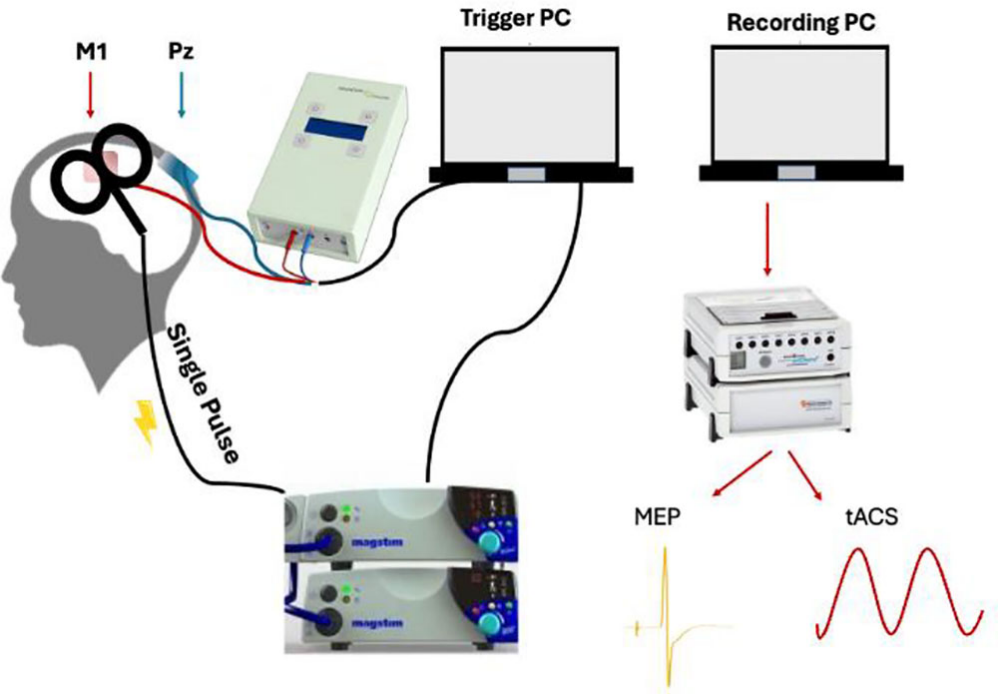
Experimental design. The tACS electrode was placed over the hand region of the left primary motor area M1 (as determined by TMS hotspotting), and the other tACS electrode was placed over the Pz (Nuwer 2018). Electrodes were held in place by a rubber strap, with two EMG electrodes attached over the M1 tACS electrode. The TMS coil was placed over the M1 (Adank et al. 2018). The trigger PC triggered and synchronized the tACS and TMS, whereas the recording PC measured the MEPs from FDI and tACS oscillations from the M1 electrode. MEPs, motor‐evoked potentials; tACS, transcranial alternating current stimulation.

Participants were recruited to take part in two separate sessions that each involved the application of combined tACS and TMS over the primary motor cortex. In each case, MEP amplitudes and trial‐by‐trial variability were the primary outcome measure. The order of the sessions was counterbalanced to control for potential sequence effects. The experimental setup is shown in Figure 1.

### 2.1 Participants

Data were collected from 15 right‐handed participants (10 females, mean age ± SD: 26.6 ± 5.7 years), all of whom had no history of neuropsychological disorders (Table 1). Prior to the study, each participant provided written informed consent and completed a TMS safety questionnaire either online or in person. The study protocol was reviewed and approved by an appropriate local ethical review committee (School of Psychology, University of Nottingham). All participants completed both sessions of the study, which were counterbalanced and scheduled to occur at least 3 days apart.

**TABLE 1:** Participant information, including motor threshold (MT) values recorded for session one (S01) and session two (S02).

ID	Age	Sex	MT % S01	MT % S02
HP01	24	F	70	70
HP02	27	F	67	72
HP03	24	F	75	75
HP04	34	M	76	76
HP05	23	F	72	72
HP06	33	M	72	72
HP07	19	F	68	68
HP08	28	F	70	70
HP09	18	F	76	76
HP10	37	M	74	74
HP11	34	F	76	76
HP12	28	F	56	56
HP13	25	M	70	70
HP14	22	F	56	56
HP15	23	M	54	54

### 2.2 Procedure

Participants were seated in a chair with their right arm and elbow placed in a comfortable position on the desk in front of them. EMG was recorded using disposable Ag–AgCl muscle electrodes (H124SG Covidien, 23.9 mm diameter). Electrodes were placed securely on the right hand in a belly–tendon montage targeting the first dorsal interosseus (FDI) muscle, with the ground electrode placed on the right ulna. EMG signals were amplified and bandpass filtered (10 Hz–2 kHz, with a sampling rate of 2 kHz) and then digitized using Brain Amp ExG (Brain Products, GmbH, Gilching, Germany) controlled by Brain Vision Recorder (Brain Products, GmbH, Gilching, Germany).

TMS hotspotting, thresholding, and single‐pulse stimulation were delivered using a MagStim Bistim^2^ stimulator (Magstim, Whiteland, Dyfed, UK) with a 70 mm figure‐of‐8 coil coupled to a BrainSight neuronavigation system (Rogue Research Inc., Montreal, Quebec, Canada). Neuronavigation was based on a template MRI scan (MNI‐152) to allow for accurate coil orientation and location over the left M1. Before delivering pulses to the scalp, participants had the opportunity to ask questions and feel a test pulse of TMS on the palm of their hand once the procedure was explained to them. Once ready, suprathreshold pulses were delivered to the left M1 hand area “hotspot,” defined as the location that consistently evoked the largest MEPs in the right FDI muscle. Coil orientation was maintained at 45° from the midline with current flowing in a posterior–anterior direction, which has been shown to evoke optimal MEP responses in the FDI muscle (Adank et al. 2018). These pulses were recorded on BrainSight, and once the hotspot was found, this area was landmarked to ensure this hotspot area was consistently targeted at the correct orientation throughout the experiment and allowed participants to take breaks if they requested one.

Once the hotspot was identified, this area was marked to ensure the correct placement of the tACS electrode. The tACS neurostimulator (NeuroConn, Germany) used tACS sponge electrodes of size of 5 cm × 7 cm. The tACS electrode was placed over the marked area (left M1), whereas the other electrode was placed over Pz (according to 10–10 system (Nuwer 2018)). The electrode dimension was 5 cm × 7 cm. The impedance of the electrodes was always below 10 kΩ for all the participants, which minimizes the tactile sensation. This was achieved by using conductivity gel and saline. The tACS electrodes were placed on the participants’ heads and stabilized there using rubber straps.

Following the hotspotting and the placement of tACS electrodes, motor thresholds (MTs) were determined for each participant using EMG responses from the FDI muscle. MT was defined as the TMS stimulation intensity (% maximum stimulator output, MSO) required to elicit a peak‐to‐peak MEP amplitude of 200–500 µV in approximately 5 out of 10 trials. The high MT was selected to ensure clear MEP visibility throughout the sessions.

In addition to the tACS electrodes, two Ag‐AgCl electrodes (H124SG Covidien, 23.9 mm diameter) were placed over the M1 tACS electrode using rubber straps. These electrodes were utilized to record real‐time tACS sinusoidal oscillations via the Brain Amp ExG system (Brain Products, GmbH, Gilching, Germany).

A custom MATLAB script was employed to trigger and synchronize tACS stimulation with single‐pulse TMS delivery. Each session consisted of four trials, each lasting 5 min, with optional breaks available to participants between sessions. At the onset of each trial, tACS and TMS were simultaneously triggered. TMS pulses were delivered with an inter‐trial interval of 4 ± 1 s, under two conditions: TMS‐pulse or no TMS‐pulse, randomly assigned across different tACS phases.

### 2.3 Analysis

EMG data for each participant and trial were inspected visually using custom in‐house software developed in MATLAB. Trials were excluded from further analysis if pre‐contraction of the FDI muscle, or noise levels, exceeded 50 µV during the 500 ms window preceding onset of MEP. The peak‐to‐peak amplitudes of MEPs were quantified within this software. Prior to further analysis, the recorded tACS oscillations were visually inspected to confirm the sinusoidal shape. If a cosinusoidal oscillation was recorded, it was corrected by phase‐reversing. A custom MATLAB script was used to calculate the alignment of the tACS phase to each participant's MEPs. The MEPs and their corresponding tACS phases were then visually inspected and categorized into four phases: peak, trough, falling edge, and rising edge. Median MEP amplitudes were calculated for each tACS phase (peak, falling edge, trough, and rising edge) for each participant. Inter‐trial variability was also assessed by calculating the coefficient of variation (CV) for each tACS phase (peak, falling edge, trough, and rising edge) and for each individual. The mean of individual median MEP values and CV values for all participants was used to identify differences across tACS phases and between stimulation frequencies. Effect sizes based on standardized mean differences (Cochrane's *D*) were calculated along with *t*‐tests to evaluate phase‐related differences in mean MEP amplitudes and CV values. By convention a Cochrane *D* value of 0.5 is taken to reflect a medium‐sized effect.

## 3 Results

### 3.1 Mean Number of TMS Pulses Delivered

Initial *t*‐tests confirmed that there were no statistically significant differences in the mean number of MEPs collected across the different tACS phases and stimulation frequencies (*p* > 0.05). Relevant mean number of TMS pulses delivered for the α‐tACS session were peak = 30, trough = 39, falling edge = 32, and rising edge = 29. And for the β‐tACS session, peak = 34, trough = 30, falling edge = 25, and rising edge = 29.

### 3.2 Motor Threshold

The mean MT% for Session 1 was 68.8 (7.54) %, and for Session 2 it was 69.1 (7.56) %. These means did not differ statistically (*p* > 0.05).

### 3.3 MEP Amplitude

**FIGURE 2:**
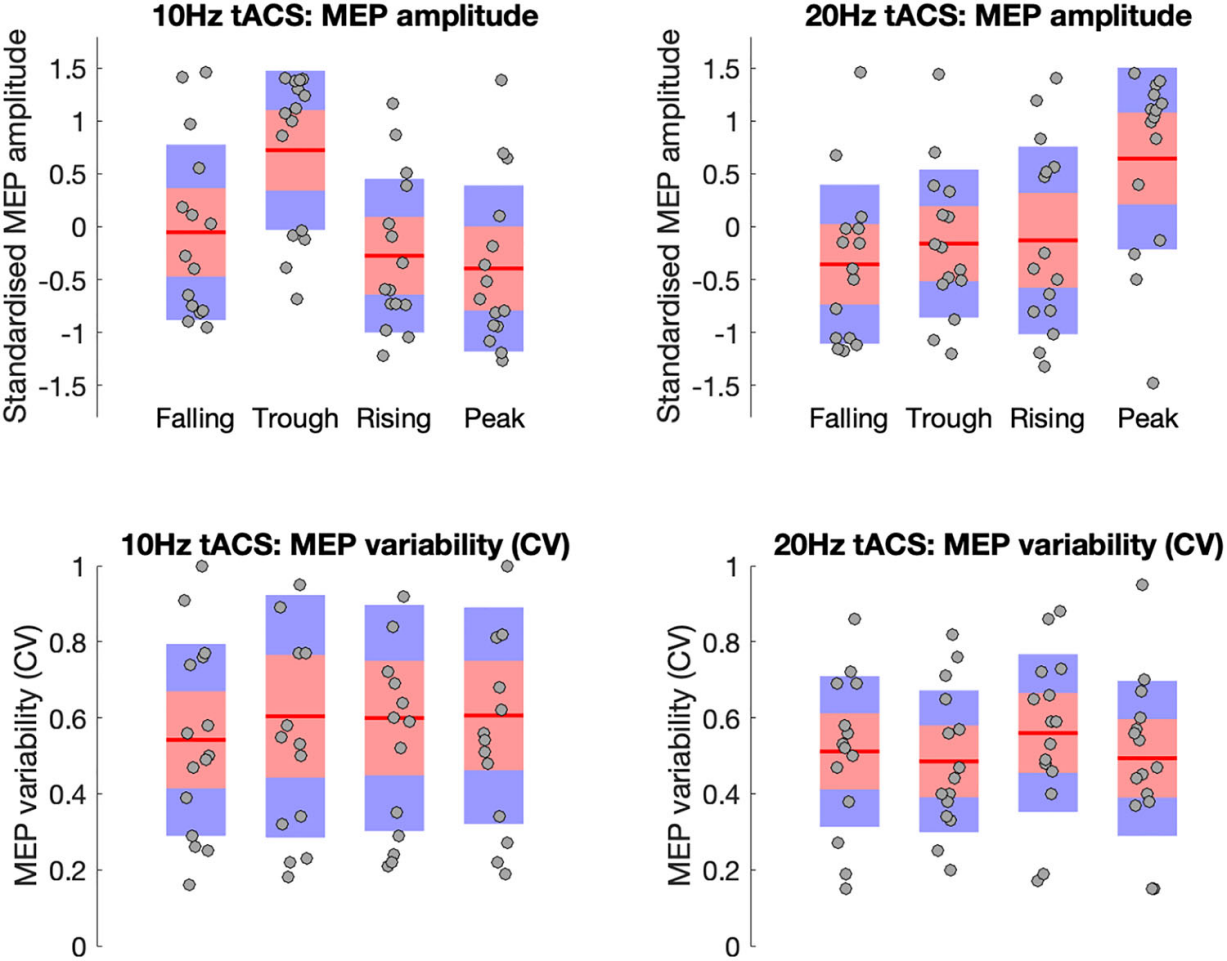
Upper panel: Boxplots showing the mean of standardized median MEP amplitudes during the α‐tACS stimulation (left) and β‐tACS stimulation (right). The grey dots indicate the individual median values, whereas the red lines indicate the group median for each condition (peak, falling edge, trough, and rising edge). Bottom panel: Boxplots showing the mean coefficient of variation (CV) values during the α‐tACS stimulation (left) and β‐tACS stimulation (right). MEPs, motor‐evoked potentials; tACS, transcranial alternating current stimulation.

Mean standardized (*z*‐score) median MEP amplitudes for α‐ and β‐tACS entrainment are presented in Figure 2 (upper panels). For α‐tACS entrainment, the mean (±SD) MEP amplitude was −0.40 (±0.8) at the peak, −0.05 (±0.8) at the falling edge, 0.72 (±0.8) at the trough, and −0.28 (±0.7) at the rising edge. Statistical analyses confirmed that when TMS was aligned with the α‐tACS trough, then MEP amplitudes were larger than when aligned with the peak, falling edge, or rising edge (minimum: Cohen *D* = 0.53; *p* < 0.05).

For β‐tACS entrainment, the mean (±SD) MEP amplitude was 0.65 (±0.9) at the peak, −0.36 (±0.8) at the falling edge, −0.16 (±0.7) at the trough, and −0.13 (±0.9) at the rising edge. Statistical analyses confirmed that when TMS was aligned with the β‐tACS peak, then MEP amplitudes were larger than when aligned with the trough, falling edge, or rising edge (minimum: Cohen *D* = 0.53; *p* < 0.05).

These findings confirm that TMS‐induced corticospinal excitability varies as a function of tACS phase. Specifically, they confirm that MEP amplitude is maximal when TMS is aligned with the trough of the α‐tACS phase cycle but with the peak when aligned with the β‐tACS phase cycle. These results are consistent with previous results obtained using EEG‐triggered TMS (Zrenner et al. 2018; Wischnewski et al. 2022).

### 3.4 MEP Variability

Mean CV values for α‐ and β‐tACS entrainment are presented in Figure 2 (lower panels). For α‐tACS entrainment mean (±SD), CV values were 0.61 (±0.3) at the peak, 0.54 (±0.3) at the falling edge, 0.60 (±0.3) at the trough, and 0.60 (±0.3) at the rising edge. Statistical analyses confirmed that when TMS was aligned with the α‐tACS falling edge, the mean CV was reduced compared to when TMS was aligned to the α‐tACS trough (*D* = −0.57, *p* < 0.05).

For β‐tACS entrainment mean (±SD), CV values were 0.49 (±0.2) at the peak, 0.51 (±0.2) at the falling edge, 0.49 (±0.2) at the trough, and 0.56 (±0.2) at the rising edge. Statistical analyses confirmed that when TMS was aligned with the β‐tACS trough, the mean CV was reduced compared to when TMS was aligned to the β‐tACS rising edge (*D* = −0.52, *p* = 0.06).

**FIGURE 3:**
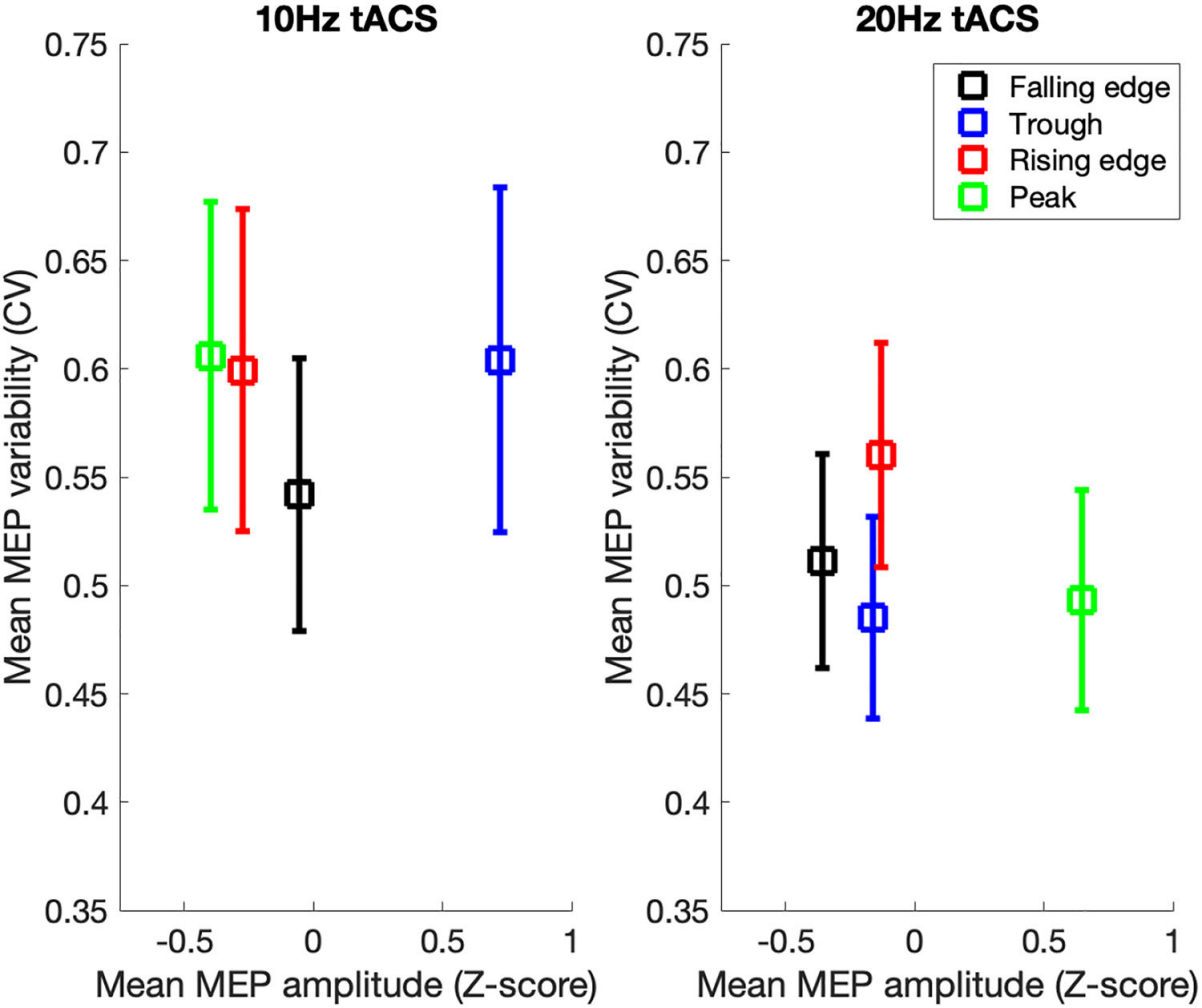
*Left panel* shows the relationship between mean TMS‐induced MEP amplitude (*x*‐axis) and mean TMS‐induced MEP variability when TMS was aligned to each phase of the α‐tACS (10 Hz) phase cycle. Error bars are the standard error of the mean (SEM). *Right panel* shows the relationship between mean TMS‐induced MEP amplitude (*x*‐axis) and mean TMS‐induced MEP variability when TMS was aligned to each phase of the β‐tACS (20 Hz) phase cycle. MEPs, motor‐evoked potentials; tACS, transcranial alternating current stimulation.

These findings confirm that trial‐by‐trial variability of TMS‐induced increases in corticospinal excitability, as indexed by CV, varies as a function of both tACS phase and tACS frequency. Interestingly, these results indicate that the optimal target for delivering phase‐aligned TMS may differ according to whether one aimed to maximize the efficacy of TMS stimulation (i.e., increase MEP amplitude) or minimize the variability of stimulation (i.e., decrease MEP CV). This can be seen in Figure 3. Inspection of this figure suggests that if one wanted to both increase efficacy of TMS and decrease variability, then one might align TMS with the falling edge of 10 Hz tACS rather than the trough.

## 4 Discussion

In this study, we used α‐ and β‐frequency tACS to entrain cortical motor oscillations, and we delivered single pulses of TMS to induce increases in corticospinal excitability as measured by EMG MEPs. Importantly, on each trial, the delivery of TMS was timed to coincide with the peak, falling phase, trough, or rising phase of the α‐ or β‐phase cycle. On the basis of prior EEG‐triggered TMS studies, we hypothesized that there would be phase‐dependent differences in the magnitude of TMS‐evoked MEPs and that these phase‐dependent effects would differ for α‐ and β‐tACS entrainment (Zrenner et al. 2018; Wischnewski et al. 2022). Importantly, we also hypothesized there would be phase‐dependent differences in the inter‐trial variability of TMS‐induced increases in MEP amplitude. Our results confirmed each of these hypotheses, which are discussed in more detail below.

It should be noted that if the positions of the anode and cathode electrodes were reversed, then the timing of the positive and negative peaks in the phase of the tACS current would be altered.

A limitation of the current study is that we did not directly observe the effects of tACS on brain activity. Instead, in the current study we have assumed that tACS delivered at a given (target) frequency can be used to entrain brain activity at that target frequency in a phase‐dependent manner. This assumption is supported by several converging lines of evidence. First, studies in non‐human primates demonstrate phase‐dependent alignment of neural spiking activity during tACS (Krause et al. 2019; Johnson et al. 2020; Lee et al. 2024). Note that in this case, the neural firing rate is not actually increased, but instead, spiking is organized to occur in bursts that are aligned with the phase of the tACS stimulation (Krause et al. 2019). Second, studies that have combined EEG recording with tACS stimulation support the proposal that tACS modulates EEG at the targeted frequency. Zaehle et al. (2010) used tACS delivered at participants’ individual α‐frequencies, and they demonstrated that α‐power was increased after tACS compared to after sham stimulation. More recently, Helfrich and colleagues overcame the inherent technical difficulties of using EEG to concurrently measure the online effects of tACS and demonstrated that 10 Hz tACS enhances EEG amplitudes in the alpha band (Helfrich et al. 2014). They also reported a narrowing of the spectral peak in the alpha band (Helfrich et al. 2014). This may indicate that, consistent with the concept of tACS phase‐aligned entrainment of brain oscillations, individual preferred α‐frequencies of participants underwent a shift toward the target 10 Hz tACS frequency. Finally, studies that have used concurrent fMRI to investigate the effects of tACS also support the proposal that tACS entrains brain activity. Specifically, previous fMRI studies have shown that α‐frequency power is negatively correlated with the fMRI BOLD signal (Yin et al. 2016; Goldman et al. 2002; Laufs et al. 2003). For this reason, Vosskuhl et al. (2016) used fMRI to investigate the effects of α‐tACS on the fMRI occipital BOLD response to visual stimulation. They demonstrated that α‐tACS reduced task‐related BOLD activation compared to tACS‐free periods (Vosskuhl et al. 2016). On the basis of these converging lines of evidence, we feel confident in concluding that tACS can be used in our study to effectively entrain 10 and 20 Hz brain oscillations.

As noted above, studies making use of real‐time phase‐aligned EEG‐triggered TMS have demonstrated that TMS‐induced increases in MEP amplitude are larger when TMS is triggered to coincide with the trough of the 10 Hz oscillation (Zrenner et al. 2018; Wischnewski et al. 2022) but, conversely, are larger when timed to coincide with the peak of the 20 Hz oscillation (Wischnewski et al. 2022). Consistent with these previous reports, we demonstrate here that TMS‐induced increases in MEP amplitude are largest when timed to coincide with the trough of the α‐tACS phase cycle or the peak of the β‐tACS phase cycle. To the extent that brain oscillations may reflect functionally important fluctuations in brain state (e.g., changes in cortical excitability), our findings, together with those of the EEG‐triggered TMS studies referred to above, highlight the impact that the frequency and phase alignment of ongoing brain oscillations may have on the efficacy of non‐invasive brain stimulation.

We hypothesized that aligning TMS with the phase of tACS might not only increase the efficacy of TMS but would also increase the consistency of the effect, by reducing trial‐by‐trial variability. Our findings demonstrate that there were indeed phase‐related differences in the trial‐by‐trial variability of TMS (as measured by CV) and that these differed for α‐ and β‐tACS stimulation. Specifically, we found that variability was reduced when TMS was aligned to the falling edge of the α‐tACS phase cycle and to the trough of the β‐tACS. This finding indicates that choosing precisely when to deliver noninvasive brain stimulation with respect to ongoing brain oscillations, it may be important to consider not only the oscillation frequency and phase, but also whether the aim is to increase the efficacy of stimulation or reduce the variability of stimulation effects.

Although this study provides evidence that tACS can be used to entrain corticospinal excitability in a frequency‐specific and phase‐specific manner, much still remains to be understood with respect to the underlying mechanisms involved. tACS is generally thought to cause the dendrites and cell bodies of cortical neurons to alter their membrane potential toward depolarization or hyperpolarization in synchrony with the oscillation frequency of the stimulation. This suggests that tACS primarily acts to organize and synchronize neural firing rather than initiate firing. Consistent with this proposal, animal studies demonstrate that although spiking becomes synchronized with the phase of tACS, the overall rate of spiking does not increase (Zrenner et al. 2018). Similarly, human fMRI studies show that although α‐tACS modulates task‐related BOLD activation compared to tACS‐free periods, there was no effect of tACS independent of the visual task (Vosskuhl et al. 2016). However, recent animal studies have demonstrated that tACS may lead to frequency‐dependent cell‐class‐specific entrainment of spiking activity (Lee et al. 2024). This raises the possibility that entrainment of cortical activity might involve the selective activation of modulatory local interneuron populations.

## Author Contributions

**Aikaterini Gialopsou**: conceptualization, data curation, formal analysis, investigation, writing – original draft, methodology, visualizations. **Stephen R. Jackson**: conceptualization, data curation, formal analysis, visualization, writing – original draft, writing – review & editing, supervision

## Conflicts of Interest

The authors declare no conflicts of interest.

## Peer Review

The peer review history for this article is available at https://publons.com/publon/10.1002/brb3.70876.

## Data Availability

The data that support the findings of this study are available from the corresponding author upon reasonable request.
